# Spatiotemporal Dynamics of Fish Density in a Deep-Water Reservoir: Hydroacoustic Assessment of Aggregation Patterns and Key Drivers

**DOI:** 10.3390/ani15071068

**Published:** 2025-04-07

**Authors:** Zihao Meng, Feifei Hu, Miao Xiang, Xuejun Fu, Xuemei Li

**Affiliations:** 1 Yangtze River Fisheries Research Institute, Chinese Academy of Fishery Sciences, Wuhan 430223, China; 17612182178@163.com (F.H.); xiangmiao@yfi.ac.cn (M.X.); 2 Aquatic Conservation and Rescue Center of Jiangxi Province, Nanchang 330096, China; fuxuejun721@163.com

**Keywords:** fish distribution, schooling behavior, environmental gradients, generalized additive models, acoustics monitoring, Zhelin Reservoir

## Abstract

Understanding spatiotemporal patterns of fish density and their environmental drivers is critical for managing river–lake ecosystems, yet dynamic interactions in heterogeneous habitats remain poorly quantified. This study combined hydroacoustic surveys, spatial autocorrelation analysis (Moran’s I), and generalized additive models (GAMs) to investigate seasonal and spatial fish distribution, aggregation characteristics, and regulatory mechanisms in China’s Zhelin Reservoir. The results reveal pronounced seasonal fluctuations, with summer fish density peaking at 13.70 ± 0.91 ind./1000 m^3^ and declining to 1.95 ± 0.13 ind./1000 m^3^ in winter. Spatial heterogeneity was evident, with the Xiuhe region sustaining the highest density (15.69 ± 1.09 ind./1000 m^3^) and persistent hotspots in upstream bays. Transient high-density clusters (90–99% confidence) near the Zhelin Dam during summer suggested thermal or hydrodynamic disturbances. GAM analysis (R^2^adj = 0.712, 78.5% deviance explained) identified seasonal transitions (12.26% variance), water depth (16.54%), conductivity (13.75%), and dissolved oxygen (13.29%) as dominant drivers, with nonlinear responses to depth and bimodal patterns for conductivity/oxygen. These findings demonstrate that hydrological seasonality and habitat heterogeneity jointly govern fish aggregation, underscoring the ecological priority of Xiuhe and upstream bays as core habitats. This study provides a mechanistic framework for guiding reservoir management, including targeted conservation, dam operation adjustments to mitigate hydrodynamic impacts, and integrated strategies for balancing hydrological and ecological needs in similar ecosystems.

## Simple Summary

Managing river and lake ecosystems requires understanding where fish are and why they are there. Fish are vital for healthy aquatic environments. This study aimed to understand how fish populations change over time and in different areas of a Chinese reservoir. Using hydroacoustic and statistical tools, we mapped fish distribution and identified the key environmental factors that influence it. We found that fish density varied greatly with the seasons and regions, with water depth, conductivity, and oxygen levels being important drivers. This knowledge can help us better manage the reservoir, protect important fish habitats, and ensure a healthy ecosystem for the future.

## 1. Introduction

Fish aggregation, the spatiotemporal organization of conspecific groups, constitutes an essential survival adaptation that enhances resource acquisition efficiency while mitigating predation risks [1]. Beyond individual fitness optimization, this behavioral strategy exerts cascading effects on ecosystem structure through trophic rewiring and energy flux modulation [2]. Although the evolutionary advantages of aggregation are well characterized, the mechanisms governing its spatiotemporal variability remain enigmatic, particularly in hydrologically dynamic systems where environmental and biotic drivers interact through scale-dependent processes [3]. Emerging evidence highlights dual regulatory controls: abiotic gradients (temperature, dissolved oxygen, light penetration) establish physiological constraints [4,5], while biotic interactions (predator–prey dynamics, resource competition) fine-tune aggregation patterns [6,7]. Crucially, these drivers exhibit non-stationary relationships across spatial scales, a phenomenon poorly captured by conventional global regression models [8]. Understanding how these factors interact to influence aggregation is a key area of the current research [9].

Conventional approaches to studying fish aggregation, including direct observation and netting surveys, are constrained by their invasive nature and limited spatiotemporal resolution [10,11]. In this context, hydroacoustic technology has emerged as a powerful non-invasive tool, enabling high-resolution monitoring of fish density and movement patterns across spatial scales [12,13,14]. Understanding the spatiotemporal dynamics of fish aggregation and their environmental drivers is crucial for effective conservation and sustainable management of these resources. When integrated with Exploratory Spatial Data Analysis (ESDA), this approach can reveal complex spatial autocorrelation patterns that traditional methods often overlook [15]. Notably, while ESDA has been extensively applied in marine ecosystems, its potential remains underexploited in freshwater systems. The interplay between fisheries and environmental drivers is inherently complex [16], exhibiting non-linear and non-additive effects, with impacts often contingent on the scale of observation [17]. To address the inherent non-linearity of environmental drivers, generalized additive models (GAMs) offer particular advantages in capturing scale-dependent effects through flexible smoothing functions [18]. Consequently, GAMs have been widely employed to analyze the relationships between fishery resource density, biodiversity, and environmental factors [19,20,21]. The integration of hydroacoustics, ESDA, and GAMs thus establishes a transformative triad for analyzing multi-scale aggregation dynamics.

Deep-water reservoirs, as anthropogenically modified lentic ecosystems, present unique challenges for aggregation ecology. Their stratified thermal regimes and regulated hydrology create distinct habitat templates compared to natural lakes [22]. Climate change amplifies these complexities, with projected warming altering mixing regimes and oxygen profiles—key aggregation determinants [23]. Although substantial research has focused on natural lakes and rivers, the mechanisms governing fish aggregation in deep reservoirs remain enigmatic due to their distinct thermal stratification patterns and anthropogenic modifications. The Zhelin Reservoir, located in the middle Yangtze River basin, exemplifies such systems, serving vital functions in flood control, water supply, and biodiversity conservation [24]. Following its designation as a no-fishing zone for the 10-year period from January 2022, this system provides an unprecedented natural experiment to study baseline aggregation patterns free from fishing pressure. However, the existing studies primarily focus on species inventories [25], leaving critical gaps in our understanding of post-ban aggregation dynamics. Building on four-season hydroacoustic surveys (February–December 2023) combined with environmental monitoring, this study aims to: (1) quantify spatiotemporal variations in fish density through ESDA, and (2) elucidate the relative importance of environmental and biological drivers using GAMs. The novelty of the present research stems from its synergistic approach, uniquely integrating a powerful methodological triad (high-resolution hydroacoustics, spatial autocorrelation analysis (ESDA), and generalized additive models (GAMs)) within the specific context of a large, deep, anthropogenically regulated reservoir (Zhelin Reservoir), an ecosystem type where fish aggregation mechanisms remain poorly understood. Crucially, this investigation leverages the unprecedented natural experiment created by the implementation of a comprehensive 10-year fishing ban beginning in January 2022. This unique timing allows us, potentially for the first time in such a system, to characterize baseline fish aggregation dynamics and their environmental drivers largely free from the confounding pressures of fisheries exploitation. Therefore, this study offers a novel and timely contribution by addressing critical knowledge gaps concerning the fundamental ecology of fish aggregation in engineered aquatic environments under relatively undisturbed conditions, yielding findings essential for advancing ecological theory and informing evidence-based management strategies for Zhelin Reservoir and similar systems globally.

## 2. Materials and Methods

### 2.1. Study Area

**Figure 1:**
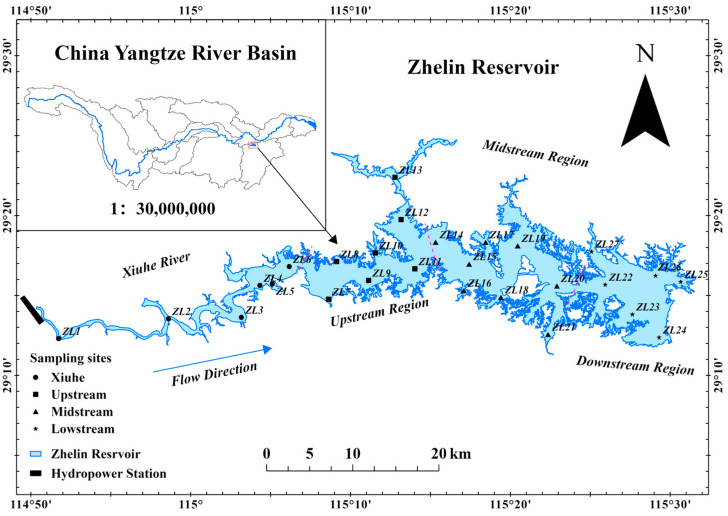
Location of the Zhelin Reservoir and sampling sites’ distribution among different regions. Inset map indicates the location of the Zhelin Reservoir in the Yangtze River Basin, China. The black line at the beginning of the image represents the Xiafang hydropower station on the Xiuhe River. The red dotted line indicates the boundary of different reservoir regions.

This study was conducted in the Zhelin Reservoir, located at coordinates 115°04′ to 115°40′ East and 29°03′ to 29°27′ North, the largest reservoir in Jiangxi Province, China, as illustrated in Figure 1. The reservoir encompasses a water surface area of 308 km^2^ and has a total storage capacity of 7.92 × 10^9^ m^3^. The average water depth is 16.3 m and the maximum depth reach 45 m. The region experiences a subtropical monsoon climate, characterized by an average annual precipitation of 1611.8 mm. Based on the geographical distribution of the Zhelin Reservoir, 27 fixed sites, extending from the Xiuhe River (ZL1~ZL6), upstream (ZL7~ZL13), midstream (ZL14~ZL21), and downstream (ZL22~ZL27) regions of the reservoir, were established to monitor abiotic and biotic factors across all seasonal campaigns (Figure 1).

### 2.2. Data Collection

Hydroacoustic surveys were conducted during the daytime in Zhelin Reservoir over the four seasons of winter (February), spring (May), summer (August), and autumn (November) in 2023. The surveys utilized a portable split-beam sounder (Simrad EK80, Kongsberg, Norway) equipped with a transducer featuring a half-power beam angle of 7°, and an operating frequency of 200 kHz. The transmission power was set to 60 W, and the pulse width was 64 µs. The transducer was mounted on the board side oriented vertically downward at a depth of about 0.5 m. The board maintained the speed of approximately 10 km/h following a zig-zag transect pattern during the survey period. Prior to surveys, the transducer was calibrated using a 13.7 mm diameter tungsten–copper sphere according to the standard calibration procedure to account for variations in the water environment [26]. The coverage degree (Dc) of the survey was conducted based on the methods of Aglen [27]. In our study, the coverage degree of four surveys with the mean values of 12.3, 11.1, 12.5, and 12.6 achieved an effective level (>6).

Both environmental and biotic factors were measured according to The Specification for Ecological Environment Monitoring of Fisheries [28] during the hydroacoustic survey. Dissolved oxygen (DO, mg/L), water temperature (WT, °C), conductivity (Cond, µS/cm), and pH of surface water (0.5 m below the surface) were measured on site using a portable multi-parameter water quality meter (HQ40D, Hach, Loveland, CO, USA). Secchi disk transparency (Tran, cm) and water depth (WD, m) were determined using a Secchi disk (SD20, Shjingmi Ltd., Shanghai, China) and Depthmate Portable Sounder (SM-5, SpeedTech, Fairfield County, CT, USA), respectively. Other environmental factors, such as total nitrogen (TN, mg/L), total phosphorus (TP, mg/L), phosphate (PO_4_, mg/L), nitrite (NO_3_, mg/L), nitrate (NO_2_, mg/L), and ammonium nitrogen (NH_4_, mg/L), were subsequently analyzed with 1 L of a water sample collected at each sampling site. Another 1 L of the water sample was filtered through glass-fiber filters (GF/F 1825-047, Whatman, Maidstone, UK) for measuring Chlorophyll-a (Chla, µg/L). For biotic factors, phytoplankton (P_Bi, mg/L) was determined from 1 L water samples subjected to 48 h sedimentation. After attaining a siphon concentration to 30 mL, subsamples (0.1 mL) were analyzed using a Sedgewick Rafter counting chamber under a compound microscope (Eclipse E200, Nikon, Tokyo, Japan) at 10–40× magnification. Taxonomic identification and abundance calculations followed Utermöhl’s method [29], with biovolume converted to biomass using standard geometric approximations [30]. Zooplankton (Z_Bi, mg/L) was quantified by filtering 10 L of water through a 100 μm mesh net (Hydrobios Kiel Net), with concentrated samples fixed in 4% formaldehyde. Taxonomic enumeration was performed in triplicate 1 mL subsamples using a Palmer counting cell under 400× magnification. Biomass estimation incorporated length-weight regressions for dominant taxa [31].

### 2.3. Statistical Analyses

Hydroacoustic data were processed using Sonar5-Pro Professional analysis software with a standardized analytical workflow. Advanced signal processing techniques, including cross-correlation filtering, were employed to mitigate interference noise and improve target detection in low signal-to-noise ratio (SNR) environments. Manual filtering protocols were systematically applied to eliminate spurious echoes originating from lithic fragments, vegetative debris, and other inorganic objects. To address acoustic interference from weak scatterers, such as plankton and crustaceans, the target strength (TS) threshold was set between −70 dB and −30 dB based on established hydroacoustic detection criteria [24,32,33]. Furthermore, a combination of automated detection algorithms and manual verification procedures ensured precise echo classification. Key operational parameters for single-target echo detection are provided in Table S1. Fish density (ρ, ind/m^3^) was computed using the volumetric method [34]:

$$v=\frac{1}{3}\pi \;\tan^{2}\frac{\theta }{2}\left(R^{3}-r^{3}\right)$$

where *v* (m^3^) represents the volume of signal unit, *θ* is the angle of the echo sounder 7°, and *r* (m) and *R* (m) are the starting and ending distance of the sounding beam, respectively. The density calculation is shown by:

$$\rho =n/\sum_{i=k}^{i=1}v_{i}$$

where ρ is the survey area fish density, *n* is the total count of valid fish echoes, *i* and *k* are the *i*th unit and total number of units, respectively.

The conversion between target strength (TS, dB) and total length (TL, cm) for fish was conducted using the empirical TS-TL formula for swim bladder fish established by Foote [35].

TS = 20lgTL − 71.9

where TS is the target strength of the fish, TL is the total length of the fish, and 71.9 is a constant.

Non-parametric Kruskal–Wallis tests were conducted to evaluate seasonal and regional differences in fish density (α = 0.05). Post hoc pairwise comparisons were performed using the Dunn test [36] to identify specific significant differences between groups. Based on the estimated fish density and central latitude–longitude coordinate data of each statistical unit, the geostatistical framework was established by constructing a raster-based spatial distribution model of fish density using Ordinary Kriging [37]. Spatial-temporal clustering patterns were analyzed through Environmental Systems Dynamics Analysis (ESDA) incorporating global and local spatial autocorrelation metrics. The former was assessed using the Global Moran’s I index (Moran’s I), ranging from −1 to 1, which quantifies the overall tendency for values to cluster (I > 1), disperse (I < 0), or exhibit a random distribution (I = 0) [38]. The statistical significance of the Global Moran’s I was evaluated was evaluated using a Z-score and associated *p*-value, following the procedures outlined by Cliff and Ord [39]. To identify the local spatial distribution of fish density, Local Indicators of Spatial Association (LISA), specifically the Getis–Ord Gi* index (Gi*) were applied to detect spatial clusters of statistically significant high-value (hotspots) and low-value (cold spots) areas [40]. The Moran’s I and Gi* indexes were calculated with the spatial analysis tools in ArcGIS Pro 3.0.2 (ESRI, Redlands, CA, USA).

To establish quantitative relationships between fish density and environmental and biotic variables, we spatially aligned fish density values extracted from GIS raster layers with corresponding sampling stations. Generalized Additive Models (GAMs) were then applied to investigate the nonlinear relationships between environmental factors and fish density trends using the ‘mgcv’ package [18]. Fish density data were log-transformed to normalize the distribution and modeled under a Gaussian error structure [41]. Before model construction, collinearity among variables was mitigated through variance inflation factor (VIF) analysis with the ‘car’ package [42], removing variables with VIF > 5 (WT: 16.0, pH: 12.6, TP: 5.1, NO3: 6.8, NO2: 7.1, Chla: 5.8) to mitigate multicollinearity. The remaining ten continuous variables and the categorical season factor were systematically incorporated into GAMs through a stepwise forward selection procedures [43]. Starting with an intercept-only null model, candidate variables were iteratively added based on their contribution to model fit, measured by the Akaike Information Criterion (AIC) [44]. In each iteration, the variable that minimized AIC (with a threshold of ΔAIC > 0) was retained. Ultimately, seven variables were incorporated into the final model, and Table 1 presents the spatiotemporal variation characteristics of these variables. The final GAM model structure is specified as:

$$\log\left(density+1\right)=\beta_{0}+s\left(WD\right)+s\left(DO\right)+s\left(Cond\right)+s\left(Z\_Bi\right)+s\left({PO}_{4}\right)+s\left(TN\right)++s\left({NH}_{4}\right)+f\left(sesaon\right)$$

where *β*_0_ is the intercept term, *s*() represents the thin-plate smoothing spline, and *f*() is the categorical factor. The variables model’s performance was validated through residual diagnostics (normality, homoscedasticity) and significance testing of individual smooth terms based on F-tests (*p* < 0.05). We estimated the relative contribution of each variable using the ‘gam.hp’ package that performs a hierarchical partitioning of the deviance explained of GAM models [45]. Data visualization was implemented using ggplot2 3.5.1 [46]. All statistical analyses were performed in R 4.4.2 [47].

## 3. Results

### 3.1. Spatiotemporal Patterns of Fish Density

**Figure 2:**
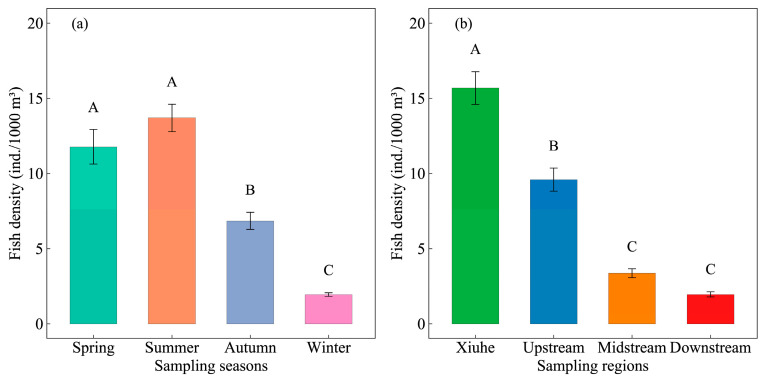
Spatiotemporal variations in fish density (ind./1000 m^3^) in Zhelin Reservoir. Panel (**a**) and (**b**) represent the seasonal and regional variations, respectively. Bars represent mean values, and error bars indicate standard error (SE). Bars with the same letter are not significantly different, while different letters indicate significant differences (*p* < 0.05) based on Dunn’s post hoc tests following Kruskal–Wallis tests.

The fish density exhibited significant spatiotemporal variation, ranging from 0.00 to 253.04 ind./1000 m^3^ (mean ± SE: 8.47 ± 0.41 ind./1000 m^3^) across the study period. Temporal analysis revealed a pronounced seasonal pattern (Figure 2a): summer achieved the highest density (13.70 ± 0.91 ind./1000 m^3^), followed by spring (11.78 ± 1.15 ind./1000 m^3^), autumn (6.85 ± 0.58 ind./1000 m^3^), and winter that showed the lowest (1.95 ± 0.13 ind./1000 m^3^). Both spring and summer exhibited significantly higher densities than autumn and winter (*p* < 0.05), although no significant difference was detected between spring and summer (*p* > 0.05). Notably, winter density was markedly lower than all other seasons (*p* < 0.01). Spatially, the Xiuhe region had the highest density (15.69 ± 1.09 ind./1000 m^3^), significantly exceeding those in upstream (9.59 ± 0.77 ind./1000 m^3^), midstream (3.36 ± 0.29 ind./1000 m^3^), and downstream regions (1.95 ± 0.18 ind./1000 m^3^, *p* < 0.001). A clear longitudinal gradient was evident (upstream > midstream > downstream), with midstream and downstream regions showing significantly lower densities than upstream and Xiuhe (*p* < 0.05), although no significant difference existed between midstream and downstream (*p* > 0.05).

**Figure 3:**
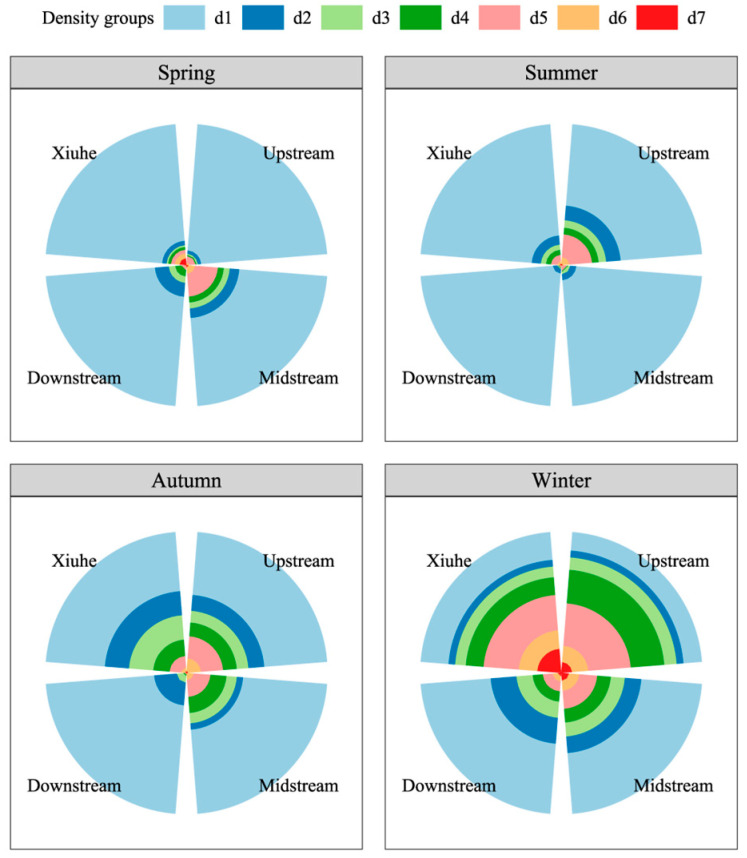
Spatiotemporal heterogeneity of fish density composition classified by target strength (TS, dB). Gradient colors represent TS groups, d1: (−70, −60), d2: [−60, −55), d3: [−55, −50), d4: [−50, −45), d5: [−45, −40), d6: [−40, −35), and d7: [−35, −30]. Sector areas represent normalized percentages of each density group.

The analysis revealed distinct spatial and temporal patterns in fish density components (Figure 3). As for the Xiuhe region, d1 maintained a stable high proportion (79.06–82.79%) from spring to autumn, significantly higher than other regions. Notably, d7 reached an exceptional winter peak of 17.01%, exceeding the inter-region average (1.53–7.59%) by over 10-fold. Meanwhile, the upstream region demonstrated pronounced seasonal variability, d5 peaked at 30.15% in winter, representing the highest annual value observed, while d4 attained its maximum winter value of 23.87%, significantly surpassing other seasons (0.47–11.46%). Seasonally, summer was dominated by high d1 values (>85%) in both downstream and midstream regions (94.07% and 89.41%, respectively), whereas d5 showed a seasonal reversal upstream, dropping to 15.98% in summer compared to its winter peak (30.15%). Winter exhibited extreme values across all regions, d5 was in the range of 6.97–30.15%, with upstream reaching 30.15%, and d4 peaked at 23.87% upstream, marking the highest annual concentration for this indicator.

### 3.2. Fish Spatial Aggregation Characteristics

The results of global spatial autocorrelation analysis for fish density are presented in Table 2. Moran’s I values remained extremely high (0.9953–0.9988) in all seasons, indicating the strong positive spatial clustering of fish density. Corresponding Z-scores (623.80–625.83) and *p*-values (all <0.001) further confirmed the statistical significance of these findings at the 0.01 confidence level. The consistent clustered distribution pattern observed in all seasons suggested that fish density exhibits stable spatial dependence throughout the year.

**Figure 4:**
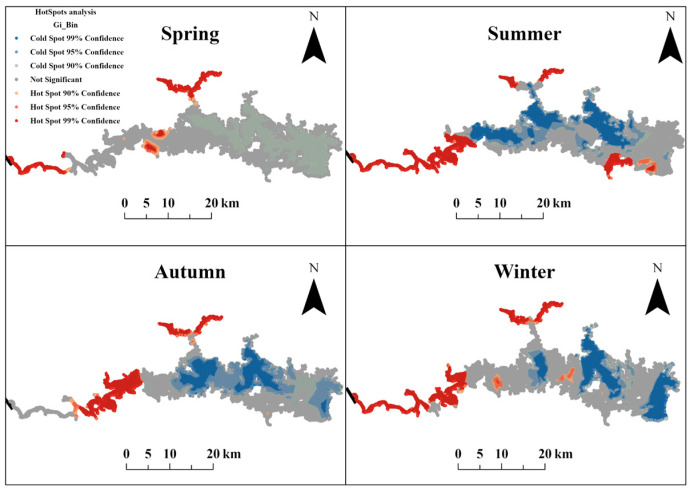
Spatiotemporal distribution of the hot- and cold spots of fish density in Zhelin Reservoir.

The analysis revealed dynamic seasonal shifts in fish density hotspots (red) alongside stable spatial patterns (Figure 4 and Table 3). Spatial analysis demonstrated that fish distribution hotspots were consistently concentrated in the Xiuhe region and the Hongyantan bay upstream, whereas cold spots prevailed in mid-lower reaches. A notable exception occurred in summer, with statistically significant hotspots with 90%~99% confidence detected near the Zhelin Dam in the downstream region, suggesting potential thermal or hydrodynamic influences. Seasonally, cold spots dominated areal proportions in autumn (61.12%), summer (56.84%), and spring (49.43%). Winter diverged from this pattern, as non-significant zones accounted for the largest share (42.81%), marginally exceeding cold spots (41.68%). Hotspots coverage remained limited across all seasons, peaking in summer (15.73%) and winter (15.51%), followed by autumn (13.54%) and spring (9.38%).

### 3.3. Key Driving Variables in Fish Distribution

**Figure 5:**
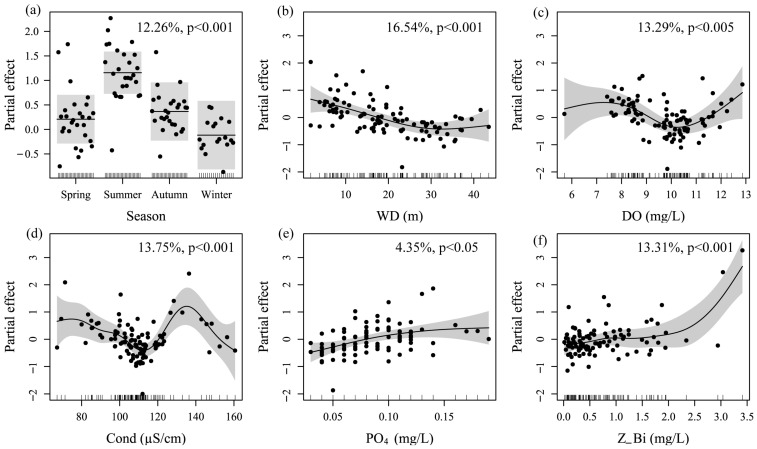
Generalized additive model (GAM) partial effect and explained deviance of selected explanatory variables on the exponential of fish density. Panel (**a**–**f**) represent season (**a**), WD (**b**), DO (**c**), Cond (**d**), PO_4_ (**e**), and Z_Bi (**f**) on fish density, respectively. Data points represent partial residuals. Tick marks on the *x*-axis indicate observed data points. Shaded areas represent 95% confidence intervals.

Eight key variables were identified in the optimal model with AIC = 196.4, R^2^_adj_ = 0.712, and 78.5% of deviance explained (Table S2). F-tests results show that all variables except for TN and NH_4_ exhibit significant nonlinear relationships with fish density (Table 4, *p* < 0.05). Seasons emerged as a critical categorical variable, explaining 12.26% of the total variance and displaying marked seasonal oscillations: summer densities peaked significantly, while winter densities reached minimal values (Figure 5a). Among environmental factors, WD formed a U-shaped curve, with moderate depths (10~15 m) representing optimal habitats and contributing the highest variance of 16.54% (Figure 5b). Cond and DO exhibited bimodal relationships, peaking at ~110 μS/cm and ~10 mg/L, accounting for 13.75% and 13.29% of the total variance, respectively (Figure 5c,d). PO_4_ showed a weak positive correlation, explaining only 4.35% of the total variance (Figure 5e), while Z_Bi exhibited a strong positive association, contributing 13.31% of the total variance, highlighting its role in reflecting community structure or trophic status (Figure 5f).

## 4. Discussion

Fish density in the Zhelin Reservoir exhibited significant spatiotemporal heterogeneity, a phenomenon resulting from the integrated effects of multiple ecological processes, including abiotic environmental filtering, habitat heterogeneity, and biotic interactions, aligning with the theoretical framework of community assembly in ecology [48,49]. As poikilothermic organisms, fish physiology is markedly influenced by ambient water temperature [50]. Temperature modulated fish metabolic rates, activity ranges, and the availability of food resources, thereby driving seasonal variations in fish density [51]. Specifically, fish density peaked during the warmer spring and summer months, while exhibiting a pronounced decline in winter, a seasonal pattern corroborated by studies in other temperate reservoirs [8,52]. Spring and summer (typically May–July) constituted the reproductive season for most fish species. Elevated temperatures and increased photoperiods enhanced metabolic activity, promoting gonadal development and spawning [53]. The subsequent increase in larval and juvenile fish abundance, depicted in Figure 3, where smaller individuals (d1 group) were proportionally more abundant, directly contributed to elevated fish densities. Furthermore, warmer temperatures favored rapid zooplankton growth, providing ample food resources for fish populations, particularly filter-feeding species [54]. The research indicates that the spring bloom of zooplankton coincides with the first-feeding period of silver carp (*Hypophthalmichthys molitrix*) larvae, significantly enhancing larval survival rates [55]. Conversely, low winter temperatures suppressed fish physiological functions, leading to reduced metabolic rates, decreased feeding activity, and a corresponding decline in fish density [56]. Moreover, wintering behavior may have influenced fish distribution, with individuals tending to aggregate in sheltered habitats, such as submerged rock piles or underwater caves, to minimize energy expenditure and evade predation [57]. This aggregation not only affected the accuracy of hydroacoustic surveys but may also have contributed to the underestimation of fish densities during winter months. It is noteworthy that the hydroacoustic methodology employed here has been rigorously validated against traditional net sampling in both our prior work [24] and other studies [8,58,59], demonstrating consistent estimates of fish size distribution and density trends. While winter aggregation behavior may introduce localized bias, the overall spatiotemporal patterns we report align with known ecological drivers, reinforcing the reliability of acoustic surveys for reservoir-scale monitoring.

The spatial distribution of fish was closely linked to aquatic habitat characteristics and surrounding environmental conditions. Our findings reveal that the Xiuhe River region exhibits the highest fish densities, with a decreasing trend observed from upstream to downstream. This spatial gradient may have been attributed to the greater habitat diversity and enhanced hydrological connectivity of the upstream waters [60]. Compared to other areas, the Xiuhe River region typically featured moderate water depths and higher primary productivity, providing ideal spawning, nursery, and refuge habitats for fish [61]. This observation aligns with numerous studies demonstrating the positive influence of a complex aquatic vegetation structure on fish abundance, diversity, and recruitment [62,63]. Furthermore, the Xiuhe River, characterized by higher hydrological connectivity, was more conducive to the reproduction of fish species that produce drifting or adhesive eggs, with increased larval abundance likely contributing to the higher fish densities observed in this region [24]. This phenomenon mirrors the findings from Amazonian reservoirs, where fish biomass is typically higher in tributary embayment compared to open water areas [64].

Conversely, upstream river reaches, influenced by riverine inputs, may have supported more efficient food webs due to higher nutrient loading and allochthons organic matter inputs [65]. In contrast, mid- and downstream regions may have exhibited less favorable conditions for survival and reproduction due to increased water depth, nutrient sedimentation, or altered hydrodynamic regimes [66]. Habitat characteristics also influenced fish community composition [67]. The upstream area was characterized by relatively uniform land use, primarily consisting of agricultural land and forests, coupled with higher aquatic vegetation cover; these more natural habitat conditions provided more favorable conditions for fish habitation and growth [68]. Compared to the upstream area, the land use patterns surrounding the mid- and downstream regions were more diverse. The downstream area encompassed the core of the Lushanxihai National Scenic Area, experiencing frequent tourist activities during major holidays, with disturbances from speedboats and recreational vessels potentially impacting fish distribution and behavior. Furthermore, the abundance of islands and more complex underwater substrate conditions in the downstream area may have favored certain fish species (e.g., mandarin fish), but also increased predation risk for smaller fish, thereby affecting overall fish density [69]. In summary, this longitudinal gradient underscores the importance of considering spatial heterogeneity, nutrient dynamics, and hydrological connectivity in studies of fish distribution within reservoir ecosystems.

Fish density exhibited significant spatial autocorrelation (significant Moran’s I value), indicating that its distribution was non-random and driven by ecological processes and spatial dependence [70,71]. Recognizing that this analysis is based on aggregated data from multiple species with potentially distinct preferences, the observed spatial aggregation likely results from the interplay of several factors operating across the community. Potential mechanisms contributing to this overall non-random pattern include habitat heterogeneity, resource availability, and social behavior [72]. Specifically, while various fish species prefer distinct environmental characteristics, spatial variations in habitat features, such as suitable water depth, substrate type, cover availability (e.g., woody debris, vegetation], and current velocity, and resource patches inevitably create a mosaic landscape [73,74]. Areas offering broadly favorable conditions, or conditions particularly suitable for dominant species, would manifest as locations of higher total fish density. Furthermore, social behaviors like shoaling, common among many fish species, can also enhance spatial clustering [72]. Similar findings have been reported in spatial autocorrelation studies of fish distribution in other aquatic ecosystems [75,76], further emphasizing the importance of spatial dependence in ecological analysis and management strategies. The identification of hotspot areas provided crucial information for targeted management interventions and conservation efforts [40]. Spatial analysis revealed that the Xiuhe River region and upstream embayment consistently represented fish density hotspots, while the mid- and downstream regions were characterized as cold spots, suggesting that the Xiuhe River region and upstream embayment should be considered critical habitats and priority conservation areas. Conversely, the mid- and downstream regions, characterized by cold spots (accounting for 61.12% in autumn), may have been subject to habitat degradation, pollution, or altered flow regimes, rendering them less suitable for fish survival [62,77]. However, the emergence of a hotspot near the Zhelin Dam in the downstream area during summer was a notable exception, potentially linked to turbulence generated by summer water releases, which can increase dissolved oxygen levels and attract hemophilic fish species [78]. Our field measurements confirmed that this phenomenon was associated with elevated dissolved oxygen levels (mean DO at hotspot stations ZL20-ZL21: 9.32 mg/L vs. 8.39 mg/L at non-hotspot stations ZL24-ZL26). The increased proportion of non-significant areas in winter (42.81%) may have been related to fish dispersal behavior during the overwintering period [79]. Seasonal shifts in fish density hotspots were influenced by both environmental changes and the alteration in natural flow regimes by dam operations [80], a phenomenon documented in other dammed systems [63,81]. Therefore, adaptive management strategies aimed at maintaining ecosystem integrity require a careful consideration of the ecological impacts of dams in reservoir ecological regulation. This study further underscores the pivotal role of seasonal and spatial variability in shaping fish distribution patterns in freshwater ecosystems [82].

Generalized additive model identified key environmental variables that significantly influenced fish density in the Zhelin Reservoir, elucidating the underlying ecological mechanisms driving fish distribution and community assembly. Seasonality explained 12.26% of the variance, reflecting the direct regulation of fish behavior and physiology by temperature and photoperiod [56]. However, environmental and biotic variables exerted a more pronounced influence on fish density compared to seasonal factors. For instance, water depth accounted for the highest proportion of variance among all variables, reaching 16.54%. the research suggests that water depths around 10 m exert the greatest influence on fish density, potentially due to higher light penetration, thermal stability, and more suitable habitat conditions [73]. Water depths less than 30 m exhibited a negative effect on fish density, potentially because fish tend to inhabit shallower areas with abundant food resources and fewer large predatory fish [83], partially explaining why fish densities were typically higher in the shallower Xiuhe River and upstream embayment compared to the deeper mid- and downstream regions. Moreover, fish density in the Zhelin Reservoir exhibited a bimodal distribution with both conductivity (Cond) and DO, peaking at approximately 110 μS/cm and 10 mg/L, respectively, potentially indicating threshold effects related to fish physiological tolerance and habitat suitability [84]. Conductivity reflects the ionic concentration of the water, potentially influencing fish osmoregulation. Different fish species exhibit varying tolerances to conductivity, potentially leading to the presence of species adapted to either low or high conductivity regions, resulting in a bimodal distribution [85]. Dissolved oxygen directly affects fish respiratory metabolism, with low and high oxygen environments potentially favoring different fish species, such as hypoxia-tolerant benthic fish and active swimming fish requiring high oxygen levels [86]. Fish density was strongly correlated with zooplankton biomass (Z_Bi), with fish abundance, particularly of smaller individuals, increasing with increasing zooplankton biomass [87], consistent with our findings. The significant positive correlation between fish density and zooplankton biomass, accounting for 13.31% of the variance, second only to water depth and conductivity, highlights the role of nutrient cycling dynamics and community structure in regulating fish density [88]. Notably, PO_4_, TN, and NH_4_ explained a relatively low proportion of the variance (less than 5%), potentially due to several factors, including the limited variability of these parameters within the study area, complex interactions with other environmental factors, or a more direct influence of other forms of nitrogen or nutrient enrichment on algal growth and food web dynamics [89]. The findings of this study further corroborate the regulatory role of water quality, habitat structure, trophic interactions, and nutrient stoichiometry on fish communities in reservoir ecosystems [90]. Although the percentage of variance explained by the model (78.5%) indicates that it provides a robust explanation for the observed patterns in fish density, additional factors, such as interspecific interactions (competition, predation), historical events (floods, droughts), and anthropogenic disturbances (pollution, habitat alteration), should be considered, as they also play a significant role in shaping fish distribution patterns [91]. Future research could further explore the influence of these factors on fish communities to gain a more comprehensive understanding of the complex dynamics of reservoir ecosystems.

## 5. Conclusions

In summary, this study elucidated the complex interplay of environmental factors, habitat characteristics, and biotic interactions shaping fish density patterns within the Zhelin Reservoir. Our findings reveal significant spatiotemporal heterogeneity in fish distribution, driven primarily by seasonal temperature fluctuations, hydrological connectivity, and habitat structure, particularly in the Xiuhe River region. The generalized additive model identified water depth, conductivity, and zooplankton biomass as key predictors of fish density, highlighting the importance of nutrient cycling and trophic dynamics in regulating fish communities. These results underscore the critical role of spatial heterogeneity and seasonal variability in structuring freshwater ecosystems and provide valuable insights for adaptive management strategies aimed at conserving fish populations and maintaining ecosystem integrity in reservoir systems. By identifying critical habitats and elucidating the ecological mechanisms driving fish distribution, this research contributes significantly to our understanding of reservoir ecology and provides a foundation for future studies exploring the impacts of anthropogenic disturbances and climate change on fish communities in similar environments. However, this study was limited by its focus on fish density as a primary metric, and future research should incorporate species-specific analyses with complementary methods, such as eDNA analysis, which is better to understand community composition and trophic interactions, and critical for developing targeted and effective fisheries management strategies. Furthermore, the relatively short duration of this study necessitates longer-term monitoring to assess the effects of internal variability and extreme events on fish populations. Future research should also investigate the role of biotic interactions, such as competition and predation, and the impacts of specific anthropogenic stressors, such as pollution and habitat alteration (e.g., benthic substrate), on fish communities in the Zhelin Reservoir and other similar systems.

## Figures and Tables

**Table 1:** Spatiotemporal variation characteristics of environmental and biotic variables incorporated into the final GAM model.

Region	Season	WD (°C)	DO (mg/L)	Cond (µS/cm)	Z_Bi (mg/L)	PO_4_ (mg/L)	TN (mg/L)	NH_4_ (mg/L)
Xiuhe	Winter	9.67 ± 7.07	10.24 ± 0.50	138.67 ± 16.37	0.30 ± 0.15	0.10 ± 0.02	1.48 ± 0.48	0.11 ± 0.10
	Spring	5.60 ± 3.21	9.75 ± 2.43	78.90 ± 10.84	0.86 ± 0.72	0.10 ± 0.02	1.31 ± 0.09	0.02 ± 0.02
	Summer	12.28 ± 6.26	9.10 ± 1.09	112.30 ± 3.95	2.24 ± 1.08	0.07 ± 0.02	0.82 ± 0.24	0.02 ± 0.02
	Autumn	11.78 ± 6.74	9.48 ± 1.19	138.88 ± 13.12	0.61 ± 0.31	0.10 ± 0.01	0.84 ± 0.10	0.03 ± 0.02
Upstream	Winter	16.08 ± 5.27	10.47 ± 0.14	111.35 ± 6.45	0.35 ± 0.23	0.08 ± 0.03	0.69 ± 0.22	0.09 ± 0.04
	Spring	13.43 ± 6.02	11.95 ± 0.57	113.79 ± 21.15	0.97 ± 0.34	0.11 ± 0.02	1.30 ± 0.16	0.01 ± 0.00
	Summer	16.60 ± 6.40	9.89 ± 0.24	111.37 ± 2.58	1.83 ± 0.30	0.08 ± 0.03	0.78 ± 0.14	0.01 ± 0.00
	Autumn	15.23 ± 5.20	8.52 ± 0.33	128.61 ± 12.24	0.54 ± 0.25	0.07 ± 0.04	0.78 ± 0.21	0.01 ± 0.00
Midstream	Winter	25.02 ± 10.13	10.48 ± 0.18	90.05 ± 7.53	0.23 ± 0.14	0.06 ± 0.01	0.55 ± 0.25	0.07 ± 0.09
	Spring	21.93 ± 9.91	11.16 ± 0.44	108.00 ± 3.79	1.09 ± 0.28	0.10 ± 0.02	1.12 ± 0.33	0.01 ± 0.00
	Summer	22.51 ± 8.00	9.89 ± 0.27	104.67 ± 2.92	0.54 ± 0.29	0.10 ± 0.06	0.82 ± 0.10	0.01 ± 0.00
	Autumn	25.70 ± 9.16	8.21 ± 0.45	113.77 ± 2.38	0.40 ± 0.10	0.08 ± 0.03	0.57 ± 0.18	0.02 ± 0.01
Downstream	Winter	29.03 ± 14.15	10.34 ± 0.17	83.60 ± 1.61	0.19 ± 0.10	0.06 ± 0.02	0.44 ± 0.02	0.02 ± 0.01
	Spring	26.30 ± 10.76	10.09 ± 0.17	109.70 ± 2.82	0.60 ± 0.37	0.09 ± 0.01	0.94 ± 0.21	0.02 ± 0.01
	Summer	28.19 ± 11.22	8.63 ± 0.28	99.44 ± 2.61	0.36 ± 0.60	0.06 ± 0.03	0.96 ± 0.16	0.02 ± 0.01
	Autumn	28.11 ± 9.96	7.85 ± 0.26	104.14 ± 2.65	0.33 ± 0.42	0.09 ± 0.06	0.62 ± 0.14	0.01 ± 0.01

**Table 2:** Global spatial autocorrelation indexes of fish density across seasons in Zhelin Reservoir.

	Spring	Summer	Autumn	Winter
Moran’s I	0.9977	0.9959	0.9988	0.9953
Z-score	625.23	623.98	625.83	623.80
*p*-value	0.000	0.000	0.000	0.000
Distribution patterns	Clustered	Clustered	Clustered	Clustered

**Table 3:** Results of hotspots analysis of fish density across seasons in Zhelin Reservoir.

Types	Spring	Summer	Autumn	Winter
Cold spots 99% confidence	0.00%	29.48%	22.49%	26.32%
Cold spots 95% confidence	0.00%	18.74%	26.46%	10.61%
Cold spots 90% confidence	49.43%	8.62%	12.17%	4.75%
No Significant	41.20%	27.43%	25.35%	42.81%
Hotspots 99% confidence	2.31%	0.86%	0.58%	1.92%
Hotspots 95% confidence	0.98%	1.54%	0.75%	2.51%
Hotspots 90% confidence	6.09%	13.33%	12.21%	11.09%

**Table 4:** Generalized additive model outputs predicting fish density from season with environmental and biotic factors.

Factor	Estimate	Standard Error (SE)	t-Value (t)	Effective Degrees of Freedom (edf)	Reference Degrees of Freedom (Ref.df)	F-Statistic (F)	*p*-Value (*p*)
Intercept (season: Spring)	1.14	0.20	5.80				<0.001
Season: Summer	0.95	0.26	3.60				<0.001
Season: Autumn	0.16	0.37	0.43				0.67
Season: Winter	−0.33	0.34	−0.95				0.35
s(WD)				2.40	3.00	6.35	<0.001
s(DO)				3.71	4.61	4.04	<0.005
s(Z_Bi)				3.81	4.69	6.29	<0.001
s(Cond)				6.14	7.25	4.23	<0.001
s(PO_4_)				1.78	2.23	5.27	<0.05
s(NH_4_)				2.26	2.75	2.36	0.10
s(TN)				1.86	2.30	0.79	0.51

## Data Availability

The data and code underlying our analysis are available on request.

## Supplementary Materials

The following supporting information can be downloaded at: https://www.mdpi.com/article/10.3390/ani15071068/s1, Table S1: Split-beam single-unit echo detection identifies specific parameters in this study; Table S2: GAM stepwise regression analysis.
