# Supplementary material for: Spatiotemporal Dynamics of Fish Density in a Deep-Water Reservoir: Hydroacoustic Assessment of Aggregation Patterns and Key Drivers
Source: Animals (Basel). 2025 Apr 7;15(7):1068. doi: 10.3390/ani15071068 (PMC11988125; doi:10.3390/ani15071068)
Supplement: Supplementary file 1 [file animals-15-01068-s001.zip › animals-3552472-supplementary.pdf]

**Table S1 Split-beam single-unit echo detection identifies specific parameters in this study**

| Parameters | Setting |
|---|---|
| Time Varied Gain,TVG | 40lgR |
| Echo Threshold | -70dB |
| Foreground Filter | [1,3] |
| Background Filter | [55,1] |
| Target Smoothing Filter | [1,3] |
| Signal Length | [3,50] |
| Maximun Gain Compensation | 6dB |
| Minimum Track Length | 2 |
| Maximun Ping Gap | 2 |
| Gating Rang | 0.3 |

**Table S2 GAMs stepwise regression analysis**

| Step | Added Variables | df | AIC | $R^2$adj | Deviance explained | Explanatory variables |
|---|---|---|---|---|---|---|
| 1 | s(WD) | 3.0 | 259.7 | 0.28 | 29.4% | WD |
| 2 | season | 6.5 | 239.4 | 0.43 | 46.4% | WD,season |
| 3 | s(DO) | 10.0 | 231.9 | 0.50 | 54.5% | WD,season,DO |
| 4 | s(Z_Bi) | 14.2 | 222.5 | 0.57 | 62.8% | WD,season,DO,Z_Bi |
| 5 | s(Cond) | 19.0 | 209.5 | 0.64 | 70.9% | WD,season,DO,Z_Bi， Cond |
| 6 | s(PO4) | 21.4 | 200.2 | 0.69 | 75.1% | WD,season,DO,Z_Bi,Cond,PO4 |
| 7 | s(NH4) | 23.4 | 197.2 | 0.70 | 77.0% | WD,season,DO,Z_Bi,Cond,PO4,HN4 |
| 8 | s(TN) | 26.0 | 196.4 | 0.71 | 78.5% | WD,season,DO,Z_Bi,Cond,PO4,HN4,TN |
